# Regorafenib treatment for patients with hepatocellular carcinoma who progressed on sorafenib—A cost-effectiveness analysis

**DOI:** 10.1371/journal.pone.0207132

**Published:** 2018-11-08

**Authors:** Amir Shlomai, Moshe Leshno, Daniel A. Goldstein

**Affiliations:** 1 Department of Medicine D and the Liver Institute, Rabin Medical Center, Petach-Tikva, Israel; 2 The Sackler Faculty of Medicine, Tel-Aviv University, Ramat Aviv, Israel; 3 Coller School of Management, Tel Aviv University, Tel Aviv, Israel; 4 Institute of Oncology, Davidoff Cancer Center, Rabin Medical Center, Petach-Tikva, Israel; 5 Department of Health Policy and Management, Gillings School of Global Public Health, University of North Carolina at Chapel Hill, United States of America; Yonsei University College of Medicine, REPUBLIC OF KOREA

## Abstract

**Background and aims:**

Hepatocellular carcinoma (HCC) is one of the leading causes of cancer related deaths. Patients with advanced HCC are treated with sorafenib. A recent randomized controlled trial demonstrated a survival benefit for regorafenib treatment in patients with advanced HCC who had progressed on sorafenib. We aimed to evaluate the cost-effectiveness of this approach.

**Methods:**

To evaluate the cost effectiveness of regorafenib, we used a Markov model that incorporates health outcomes, measured by life-years and quality-adjusted life-years (QALYs). Drug costs were based on 2017 discounted prices. Model robustness was validated by probabilistic sensitivity analyses using Monte Carlo simulations.

**Results:**

The use of regorafenib results in a gain of 19.76 weeks of life (0.38 Life Years) as compared to placebo. When adjusted for quality of life, using regorafenib produced a gain of 0.25 quality adjusted life years (QALYs). The incremental cost-effectiveness ratio for regorafenib compared with best supportive care was between $201,797 and $268,506 per QALY.

**Conclusion:**

The modest incremental benefit at a relatively high incremental cost of regorafenib treatment suggests that it is not cost-effective at commonly accepted willingness to pay thresholds.

## Introduction

Hepatocellular carcinoma (HCC) is the fifth most common cancer and the third cause of cancer-related deaths worldwide[1]. Major risk factors for the development of HCC are cirrhosis derived from any etiology and chronic infection with hepatitis C virus or hepatitis B virus[2]. Patients whose disease is diagnosed at an early stage have a good prognosis, since loco-regional radiofrequency ablation (RFA) or liver resection for limited disease or alternatively, transplantation for those with cirrhosis and portal hypertension, are all potentially curative. However, therapeutic options for patients with an advanced and/or metastatic disease are much more limited and accordingly prognosis is grave [3].

Until recently, the only FDA approved first line drug for patients with advanced HCC (stage C according to BCLC classification) is the multi-kinase inhibitor sorafenib[4]. Unfortunately, large trials have shown only a minimal benefit with sorafenib, translated to a median survival and time to progression benefit of less than three months, at the cost of potentially serious side effects[5, 6]. Therefore, until more effective and safe therapies for patients with advanced HCC are made available, one should cautiously consider the benefit of this treatment on an individual basis, especially in patients with more advanced liver disease[7].

Regorafenib is a multi-kinase inhibitor with a more potent activity compared to sorafenib[8], currently approved for the treatment of patients with advanced colorectal cancer who failed previous therapies [9]. Recently, a phase 3 clinical trial (RESORCE trial, ClinicalTrials.gov, number NCT01774344) demonstrated that patients with advanced HCC and Child-Pugh A cirrhosis whose disease has progressed on sorafenib treatment could benefit from treatment with regorafenib [10]. The median survival benefit for regorafenib treated patients in this study was 2.8 months as compared to placebo treated patients. Notably, treatment was accompanied by adverse events of hypertension, hand-foot skin reaction and diarrhea in a large proportion of patients.

Regorafenib is given in 4-week cycles in which the drug is given for 3 weeks with one week off. However, regorafenib comes at a high financial cost. Recently, we showed that treating patients with advanced colorectal cancer who failed a previous treatment, with regorafenib, results in a minimal incremental benefit at high incremental costs [11]. Given the marginal benefit of regorafenib in HCC patients shown in the RESORCE study, the incremental benefit of regorafenib relative to its potential costs in these patients should be further explored.

A recent study by Parikh et al[12] showed, based on the RESORCE trial, that sorafenib treatment as a second line is not cost-effective. However, this study assumed a constant disease progression over-time, an assumption that does not necessarily reflects the real-life situation.

Accordingly, in this study, we aimed to evaluate the cost-effectiveness of regorafenib as a second line therapy for patients with advanced HCC and Child-Pugh A cirrhosis who had progressed on sorafenib. In our analyses, we assumed that the transition probabilities between disease states depend on time.

## Materials and methods

The data from the RESORCE randomized controlled trial were obtained from a published manuscript[10]. The data is anonymous and therefore no informed consent was obtained.

**Fig 1:**
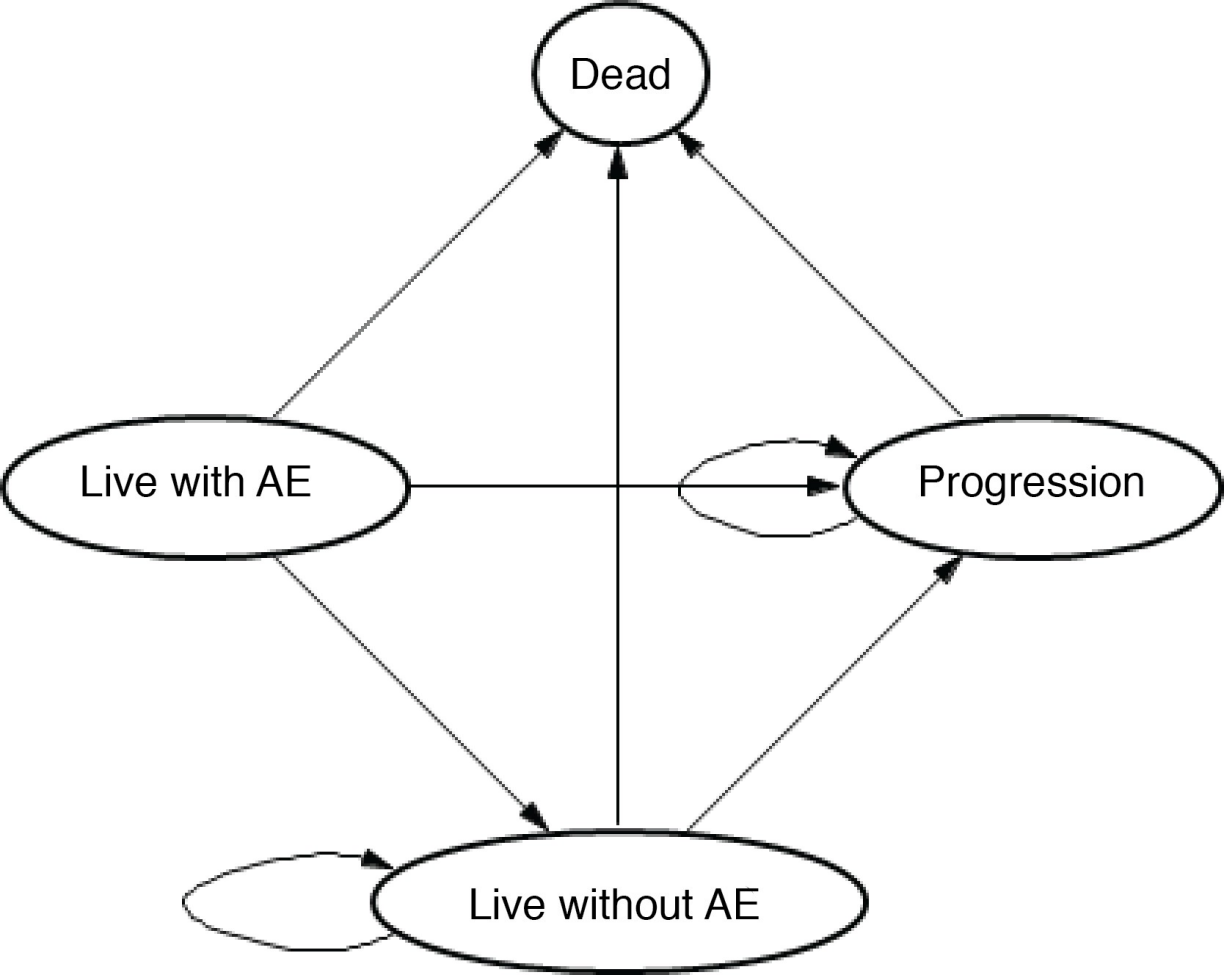
A scheme illustrating the Markov model used in this study (AE, adverse events).

We constructed a Markov model with an initial decision regarding treatment with regorafenib plus supportive care or best supportive care only. As shown in Fig 1 and described in detail previously[11], patients who initially received regorafenib could stop treatment because of either disease progression or intolerance (grades 3–4 adverse events). Patients who experienced progression after regorafenib could receive best supportive care. Progression to death could occur from each health state.

A model cycle represents daily regorafenib treatment for 3 weeks followed by a 1-week break (overall a 4-week cycle). The primary outputs of the model are as follows: life-years (LYs) and quality-adjusted LYs (QALYs), which were used to calculate the incremental cost- effectiveness ratio (ICER). The Markov model was implemented in TreeAge Pro 2018 software and statistical analyses were performed in MATLAB.

### Model survival estimates

Our assumptions were based on the survival benefits associated with regorafenib treatment in patients who had previously progressed on treatment with sorafenib, according to the RESORCE trial[10]. The overall mortality rate, which corresponded to the probability of death, was derived from the Kaplan-Meier overall survival (OS) curves for treatment with regorafenib and placebo published in the RESORCE trial. MATLAB software was used to extract the data points from the OS curves, and these data points were then used to fit parametric survival models as previously described[11]. We found that Weibull models provided a good fit for all curves (S1 Fig). On the basis of the fitted Weibull OS model, denoted as S(t), we computed the cause-specific mortality M at cycle t as: $M=1-\frac{S(t+1)}{S(t)}$

Based on Technical Support Document (TSD) 14 by the National Institute for Health and Care Excellence (NICE) Decision Support Unit (DSU), the six most common families of parametric models were assessed for best fit to the trial data as recommended by NICE[13]. These were: (1) the exponential, (2) Weibull, (3) Gompertz, (4) log-normal, (5) log-logistic and (6) generalized gamma models. The goodness of fit of the parametric models were tested visually and by comparing the Akaike Information Criterion (AIC) and the Bayesian Information Criterion (BIC), indexes which measure the adequacy of fit to the data. The Weibull distribution was the best parametric estimation of the Kaplan Meir curves.

### Progression risk

Treatment discontinuation due to AEs or progression on therapy in the regorafenib treatment group were estimated assuming an exponential distribution based on the median treatment duration published in the RESORCE trial. Estimates of mortality and progression risk beyond the follow-up time in the clinical trials were extrapolated based on the fitted survival models, as previously described[11].

### Utility estimates

Each health state was assigned a health utility score based on quality-of- life data collected in the RESORCE trial. We used a utility of 0.76 in the progression-free state and 0.68 in the case of progression. To compute the total QALYs in the Markov model, survival time was adjusted by the utility. We included grade 3 to 4 AEs in the model that had significantly different rates between the arms of the RESORCE trial, which were hand-foot syndrome, hypertension, diarrhea, and fatigue. Disutilities associated with AEs were estimated based on established values in the literature[14]. For the temporary health states associated with AEs modeled in this study (fatigue, hand-foot syndrome, diarrhea, and hypertension), the measured decreases in utilities from the published literature and the unmeasured decreases in utilities for these same health states in the RESORCE study were expected to be similar. The duration of AEs was estimated based on clinical experience. 14 days for hand-foot syndrome with a disutility of -0.116[14]. Five days for hypertension with a disutility of zero[14]. Five days for diarrhea with a disutility of -0.103[14]. Fatigue was assumed to last for 10 days, with a disutility of -0.115[14]. The duration-adjusted disutility was subtracted from the baseline utility to calculate the overall utility of each health state.

### Cost estimates

Only direct medical costs were considered and stated in 2017 US dollars. To estimate the unit price of regorafenib, we used 2017 prices from GoodRX (https://www.goodrx.com/regorafenib?drug-name=Regorafenib). Good RX is an online source for American drug prices that incorporates discounts. Regorafenib is dosed in 40-mg tablets, and the recommended starting dose is 160 mg. The price is $180.79 per 40-mg tablet. The RESORCE trial states that the mean daily dose received was 144 mg. We performed analyses in the model with three different dosing strategies: 120, 160, and 144 mg daily, with the later dosing not being realistic in clinical practice but providing an average value.

Assumptions for management of AEs were based on recently published guidelines and as previously described[11]. Clobetasol cream 0.05% and 4% lidocaine cream for the treatment of hand-foot syndrome. Amlodipine 5 mg daily for the treatment of hypertension. Lomotil and loperamide for the treatment of diarrhea. We assumed no specific medical management for fatigue. AE costs were calculated according to the Medicare physician fee schedule for 2017. Outpatient physician visits fees were based on current procedural terminology codes. The methods used for these cost calculations were previously described by Tumeh et al.[15]. We performed annual discounting of the costs and benefit in this analysis, at a rate of 3%.

### Sensitivity analysis

Internal model validations were performed, demonstrating a close approximation between the OS curves generated by the Markov model simulation and those presented in the RESORCE trial. To evaluate the robustness of the model and address uncertainty in the estimation of model parameters, a series of sensitivity analyses were performed. Utilities and drug costs were varied within ± 20% of their baseline values, in accordance with established approaches. The range for the cost of regorafenib was only -20%, as we did not expect the price to be higher than published. In univariable sensitivity analyses, we varied the value of one parameter at a time over its defined range and examined the effect on the ICER. We used the lower boundary for 120-mg dosing ($7,422 for one cycle of therapy) and the upper boundary for 160-mg dosing ($14,843 for one cycle of therapy) to provide the range of costs of regorafenib. In probabilistic sensitivity analyses, we performed 1,000 Monte Carlo trials each with 1,000 participants simulations, each time randomly sampling from the distributions for all parameters simultaneously. The baseline values, ranges, and distributions of model parameters are listed in Table 1.

**Table 1:** Model parameters: Baseline values, ranges, and distributions for Monte Carlo sensitivity analysis.

Variable	Value	Lower range	Upper Range	Reference	Distribution
Age	64	50	70	RESORCE	NA
Cost diarrhea	$81.60	$65.28	$97.92		gamma
Cost fatigue	0	0	0		gamma
Cost hand foot syndrome	$134.48	$107.58	161.38		gamma
Cost hypertension	$59.10	$47.28	$70.92		gamma
Duration diarrhea	5 days	-	-	Estimated	gamma
Duration fatigue	10 days	-	-	Estimated	gamma
Duration hand foot syndrome	14 days	-	-	Estimated	gamma
Duration hypertension	5 days	-	-	Estimated	gamma
Disutility diarrhea	-0.103	-0.082	-0.123	(12)	beta
Disutility fatigue	-0.115	-0.093	-0.139	(12)	beta
Disutility hand foot syndrome	-0.116	-0.093	-0.139	(12)	beta
Disutility hypertension	0	0	0		
γ Placebo progression	0.86233	0.8	0.9	RESORCE	Triangular
γ Placebo survival	1.07166	1.05	1.09	RESORCE	Triangular
γ Regorafenib progression	0.92149	0.9	0.95	RESORCE	Triangular
γ Regorafenib survival	1.06134	1.06	1.07	RESORCE	Triangular
λ Placebo progression	0.4345	0.4	0.5	RESORCE	Triangular
λ Placebo survival	0.0775	0.06	0.08	RESORCE	Triangular
λ Regorafenib progression	0.21159	0.2	0.25	RESORCE	Triangular
λ Regorafenib survival	0.05389	0.03	0.07	RESORCE	Triangular
Incidence diarrhea placebo	0%	0%	0%	RESORCE	beta
Incidence diarrhea regorafenib	3%	2%	4%	RESORCE	beta
Incidence fatigue placebo	5%	4%	6%	RESORCE	beta
Incidence fatigue regorafenib	9%	8%	10%	RESORCE	beta
Incidence hand foot syndrome placebo	1%	0%	2%	RESORCE	beta
Incidence hand foot syndrome regorafenib	13%	11%	15%	RESORCE	beta
Incidence hypertension placebo	5%	4%	6%	RESORCE	beta
Incidence hypertension regorafenib	15%	13%	17%	RESORCE	beta
Discount rate	0.03	0	0.05	N/A	
Utility of base	0.76	0.61	0.91	(24)	Normal
Utility of progression	0.68	0.54	0.82	(24)	Normal
Cost of Regorafenib 120 MG ($ per month)	11,389	N/A		GoodRX	
Cost of Regorafenib 144MG ($ per month)	13,667	N/A		GoodRX	
Cost of Regorafenib 160 MG ($ per month)	15,186	12,149	15,186	GoodRX	Triangular

## Results

### Base case results

Table 2 outlines the base case model results. The use of regorafenib and BSC compared with placebo and BSC resulted in a gain of 19.76 weeks of life (0.38 LYs). When adjusted for quality of life, using regorafenib produced a gain of 0.25 quality-adjusted life-years (QALYs). Accordingly, the incremental monthly cost of a course of treatment with regorafenib was between $11,410 (120-mg dosing) to $15,186 (160-mg dosing). The ICER for regorafenib compared with best supportive care was between $201,797 and $268,506 per QALY.

**Table 2:** Base case results.

Strategy	Total Incremental Cost ($) per patient	LY	Incremental LY	QALY	Incremental QALY	ICER ($/QALY)
Placebo		0.92		0.63		
Regorafenib (120mg)	50,022	1.30	0.38	0.88	0.25	201,797
Regorafenib (144mg)	60,003	1.30	0.38	0.88	0.25	242,063
Regorafenib (160mg)	66,558	1.30	0.38	0.88	0.25	268,506

### Sensitivity analyses

**Fig 2:**
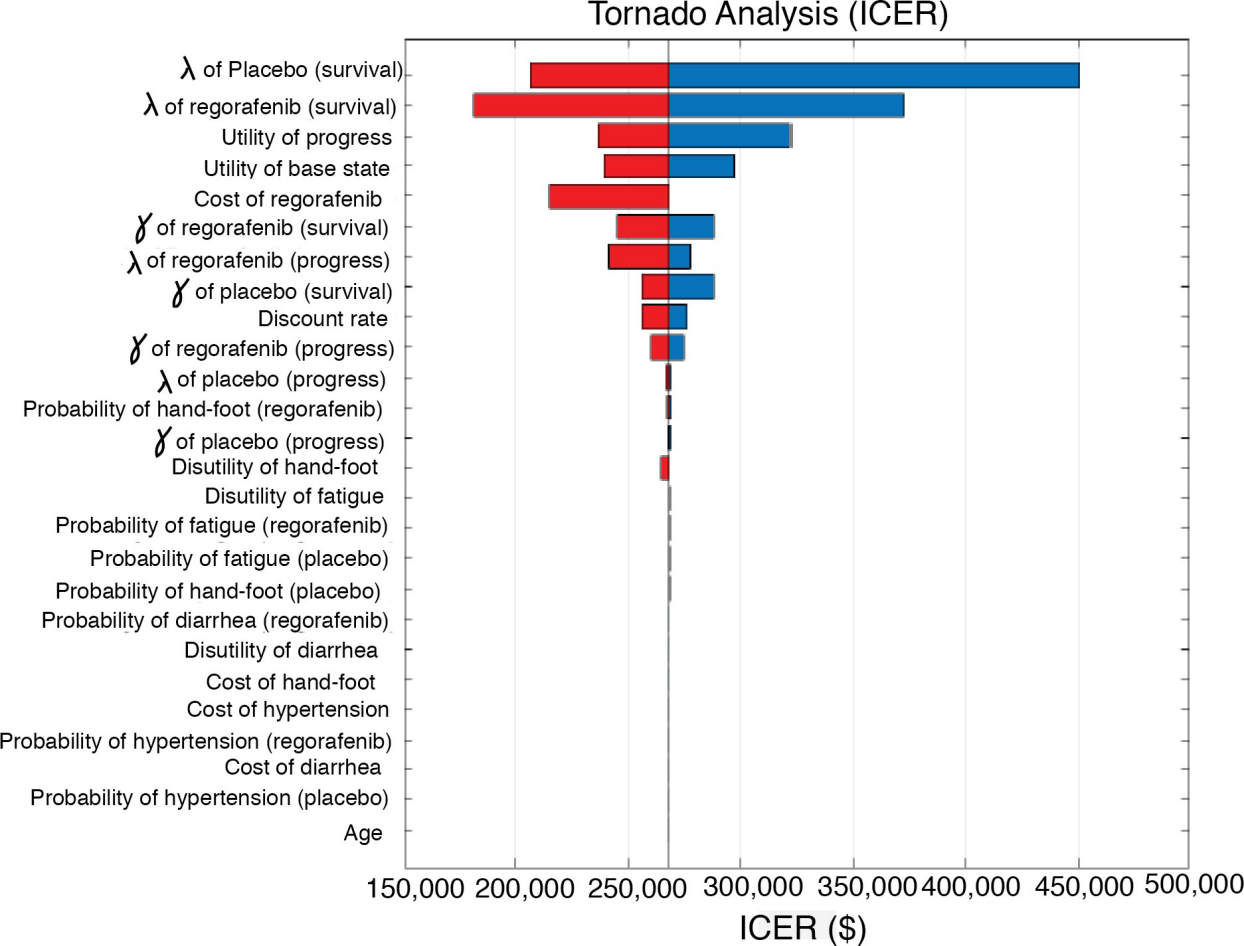
A univariable sensitivity analysis of the ICER for different parameters over the range for each parameter (ICER, incremental cost-effectiveness ratio).

The results of univariable sensitivity analyses are presented in Fig 2. Across broad variation in the ranges for each parameter, the ICER remained > $150,000 per QALY. The duration, cost, and disutility for AEs had only a minor influence on the ICER.

**Fig 3:**
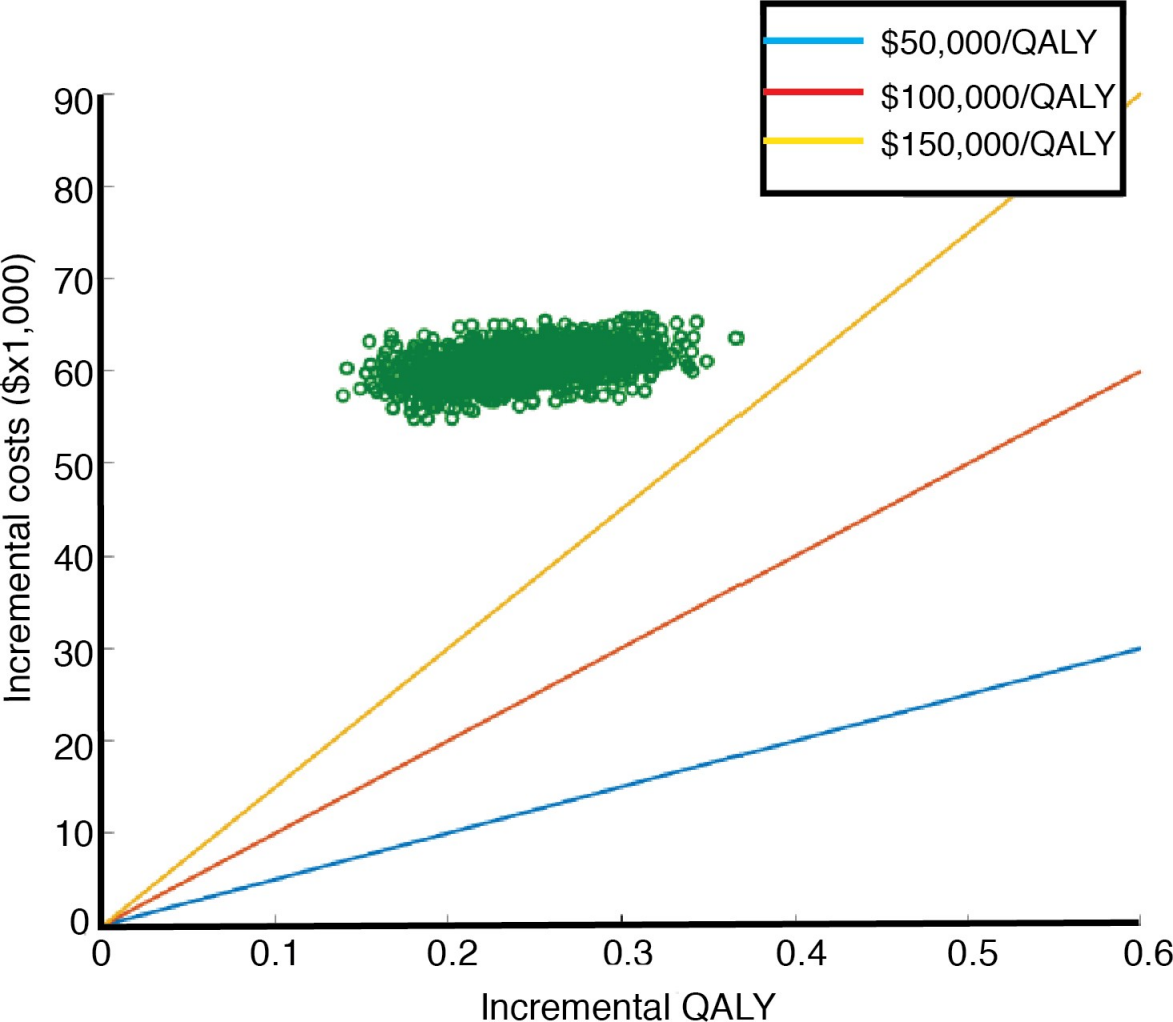
A probabilistic sensitivity analysis using the Monte-Carlo simulation plot (see details in the methods section). The lines represent three different willing to pay thresholds.

**Fig 4:**
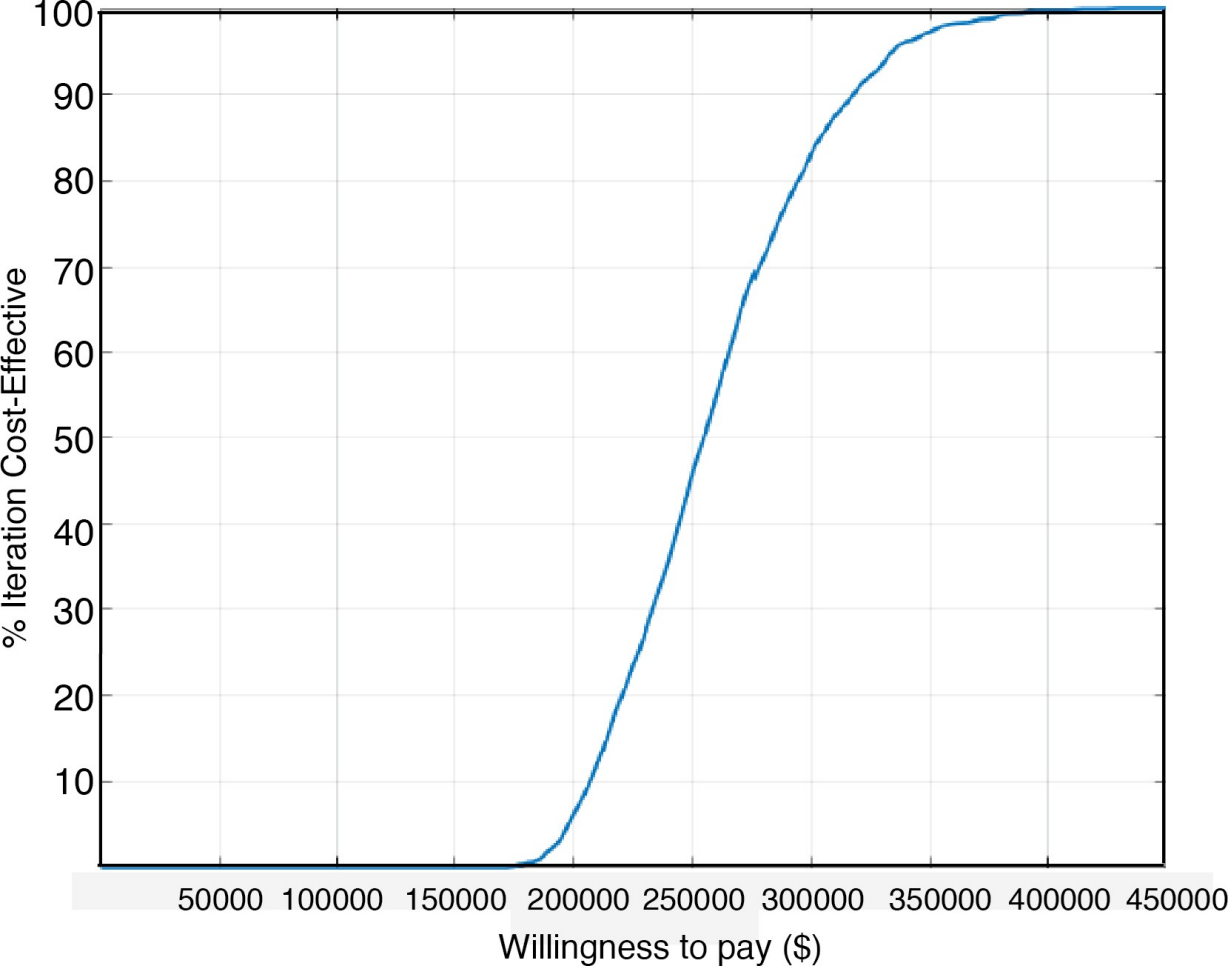
Cost-effectiveness acceptability curve for regorafenib treatment in the overall sample.

The results of the probabilistic sensitivity analyses are shown in the Monte-Carlo simulation plot (Fig 3) and in the cost-effectiveness acceptability curve (Fig 4). This curve shows the probability that regorafenib is cost-effective across increasing willingness-to-pay (WTP) thresholds. These results demonstrated 0% likelihood that regorafenib is cost-effective at WTP thresholds <$150,000 per QALY.

## Discussion

HCC is the fifth most common malignancy in men and the seventh in women and is the third leading cause of cancer death worldwide[1]. Major efforts are focused on prevention strategies in high-risk populations, including vaccination for HBV[16], anti-viral therapies for both HBV and HCV infections[17, 18] and screening programs for cirrhotic patients[19]. However, only limited progress has been made in recent years in finding more effective therapeutic approaches for patients who are already afflicted with this deadly disease[20]. One of the reasons for the slow progress in the field is the molecular heterogeneity of HCC, largely derived from the variety of etiologies leading to liver oncogenesis[21]. Furthermore, a large heterogeneity can be frequently found even in the same tumor[22], further complicating the search for a systemic drug that may target the “Achilles heel” of the tumor and thereby lead to cure.

In light of this, the introduction of the multi-kinase inhibitor sorafenib, simultaneously targeting several major pathways altered in a large proportion of liver tumors, was accompanied by great enthusiasm. However, results from large trials performed in different geographical areas around the globe, in which Child-Pugh A cirrhotic patients with advanced HCC were treated with sorafenib, provided only modest incremental benefit at a cost of potentially debilitating adverse events[5, 6].

Several studies have analyzed the cost-effectiveness of sorafenib among patients with advanced HCC, and their conclusions largely depend on the source of data they relied on. For example, a Canadian study that analyzed the results of the SHARP trial, has shown that sorafenib treatment is cost-effective as compared to best supportive care (BSC) in patients with advanced HCC. An Italian cost-effectiveness study, on the other hand, which was based on the SOFIA trial, has found that dose-adjusted, but not full-dose sorafenib treatment is cost-effective in patients with intermediate and advanced HCC compared to BSC[23]. Another study, based on the SEER-Medicare database, has found that among elderly patients with advanced HCC, sorafenib treatment results in improved survival but the same treatment is not cost-effective in patients with hepatic decompensation[24].

Until recently, patients who have progressed on sorafenib treatment had no real option for salvage therapy. The results of the RESORCE trial show that treatment with regorafenib resulted in a statistically significant improvement in overall survival compared with placebo in patients whose disease has progressed on sorafenib. Statistically significant improvement over placebo was also shown for the secondary endpoints of progression-free survival, time to progression, disease control and overall tumor response[10]. Our study investigates the cost-effectiveness of regorafenib in this group of patients, showing an incremental gain of 0.25 QALYs with an ICER for regorafenib compared with best supportive care of between $201,797 and $268,506 per QALY. Although the $50,000-per-QALY threshold for drug cost-effectiveness has been questioned recently and was suggested to be too low[25], the WTP for oncologic drugs in the US is estimated to range between $50,000-$150,000[26], well below the cost per QALY for regorafenib according to our study.

These results call into question the value of this costly treatment not only from a public point of view but also on an individual basis. The results suggest that clinicians should carefully consider the risks and benefits before treating this specific population of patients with a costly drug associated with significant adverse physical and financial impact.

Our study’s conclusion is in line with a recent study done by Parikh et al.[12], examining the cost effectiveness of regorafenib as a second line therapy for HCC. Using a Markov stimulation model, the authors have concluded that at the current price, regorafenib is not cost effective.

However, in this study the authors have assumed a constant progression rate of the disease over time, an assumption that does not necessarily reflect the real-life situation. Our study reflects the progression rate of the disease as observed in the RESORCE trial and therefore may be more accurate.

While there were some differences between our model design and the model design by Parikh et al, the overall findings were similar. Parikh at al found an ICER of $224,362 per QALY, while we found an ICER of $268,506. Both of these ICERs are substantially above commonly accepted thresholds, and provide confirmation that the ICER of regorafenib is very high. Our study provides additional support for health care payers to call for price reduction of regorafenib.

Our study has several limitations. First, for the cost-effectiveness calculations we used the estimated price of the drug in the USA and thus the applicability of our findings to other parts of the world is not straight-forward. Second, the estimated price we used may be inaccurate due to additional discounts that we are unaware of. Third, our calculations are based on results of a randomized controlled trial with a highly-selected study population that do not necessarily reflect the real-world data[27], as was demonstrated in the case of sorafenib treatment[28]. Therefore, in the future, real-world data could potentially reveal a much inferior cost-benefit estimation compared to our findings in this study.

In our study, we assumed that transition probabilities depend on time. Although transitions also depend on individual characteristics, we were limited by the available data from the RESORCE trial. For this reason, we conducted a sensitivity analysis over a large range of HR values (or equivalent parameters of the Weibull distributions) and a Monte Carlo simulation where the transition probabilities for each subject are different.

In summary, our study reveals modest incremental benefit at a relatively high incremental cost of regorafenib treatment in HCC patients who had progressed on sorafenib treatment. These findings suggest that a highly selective approach in choosing patients for this treatment is warranted.

## Supporting information

S1 Fig A comparison of the survival curves (upper panel) and the progression-free survival curves (lower panel) between the actual data and the data derived by using the Weibull model.
(TIF)

S1 File A file containing all the basic data sets and the analyses files performed in this study. (ZIP)
