# Supplementary figures and images for: Regorafenib treatment for patients with hepatocellular carcinoma who progressed on sorafenib—A cost-effectiveness analysis
Source: PLoS One. 2018 Nov 8;13(11):e0207132. doi: 10.1371/journal.pone.0207132 (PMC6224101; doi:10.1371/journal.pone.0207132)

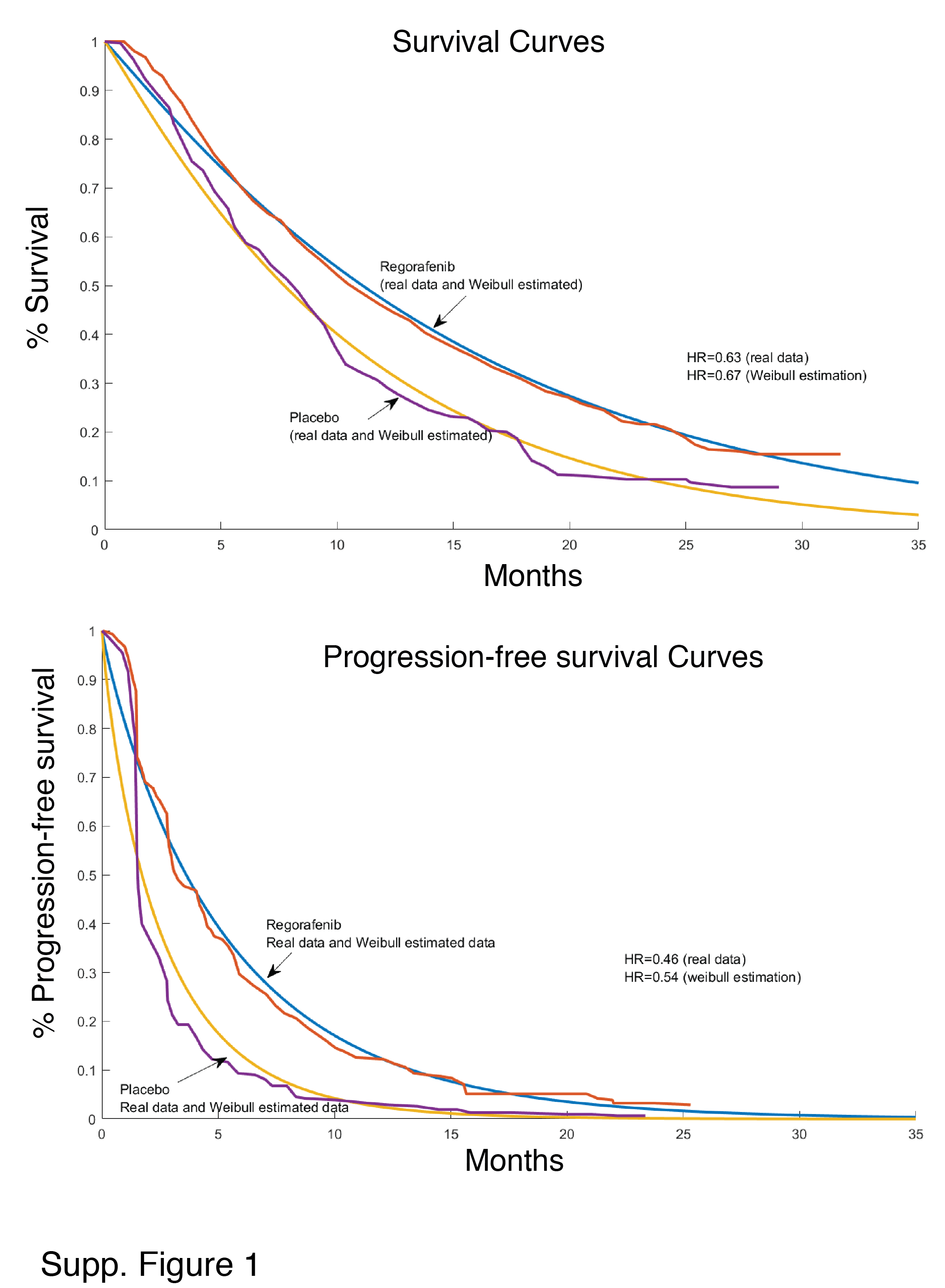

Supplement: S1 Fig — A comparison of the survival curves (upper panel) and the progression-free survival curves (lower panel) between the actual data and the data derived by using the Weibull model. (TIF) [file pone.0207132.s001.tif]
