# Supplementary material for: Regorafenib treatment for patients with hepatocellular carcinoma who progressed on sorafenib—A cost-effectiveness analysis
Source: PLoS One. 2018 Nov 8;13(11):e0207132. doi: 10.1371/journal.pone.0207132 (PMC6224101; doi:10.1371/journal.pone.0207132)
Supplement: S1 File — (ZIP) [file pone.0207132.s002.zip › S2_File/A list of the files provided-analyses.docx]

1.      Assumption file – an Excel file containing the assumption of the model

2.      Bo file – an Excel file containing the results of the Tornado analysis to run with MATLAB software

3.      CE_Dist_Final file – a TreeAge software file to run Monte Carlo simulation

4.      CE_Final – a TreeAge software file to run the Markov model

5.      CE_Final-TreeModel – an Excel file running the Markov model. This file is the result of converting a TreeAge file to an Excel file

6.      Data – an Excel file digitizing the Kaplan Meir curves in the paper for estimating the Weibull parameters

7.      HCC.m file - a MATLAB (software) file estimating the Weibull parameters of the Kaplan Meir curves in the paper.

8.      Myfunction.m file - a MATLAB (software) file necessary to run HCC.

9.      Scat.m file - a MATLAB (software) file to obtain the scatter plot

10.   Tornado.m file - a MATLAB (software) file to obtain the Tornado diagram
